# A Great Wave of Enthusiasm

**Published:** 1920-04-24

**Authors:** 


### THE MODERN UNIVERSITIES.

### A Great Wave of Enthusiasm.

All psychologists appreciate that the mental faculties of the average youth only become ripe for rapid expansion at the age of adolescence, which, for convenience, can be taken at fourteen or fifteen years; an age when the majority of the nation's youth are expected to leave school. Unfortunately for many, this is just the dawn of mature understanding. Education before this time is frequently accepted as a burden to be borne, its " Whys " and " Wherefores " being beyond the range of interested comprehension. It is also the time for the formation of stable character, because the sense of responsibility has commenced.

Secondary schools, in raising, as they have done, their standard of education, have bridged the pupil over to the period of awakening. The longer experience of mental exercise and increased information in this new condition of ripened mind and body inspires in the brightest and best pupils a zeal for fuller and more profound knowledge, which is not deterred by the severer matriculation examinations instituted by the modern universities.

### The Zeal of Earnest Youth.

Now that this wave of enthusiasm is bringing large numbers to the doors of the university it is imperative that those doors be flung wide, and that the zeal of earnest youth shall not be damped by the despairing words " No room." It is probably reasonable that the test of entry be made exacting, as it has been, so as to ensure that only men and women of capacity should take up the time and abilities of gifted professors; but once youth has filled this requirement the rest should be made easy, for on the selected or super-minds depends the Empire's strength.

It is to its fresh and vigorous youth that the Empire looks for advancement, along the lines of those inventions, discoveries, improvements and developments that keep it in the forefront of the empires of the world. It is by the development of such a class that we look for leaders and statesmen, and the permeation of public sentiment with such sound and solid knowledge that unsettling revolutionary and wildly speculative methods will be killed at their inception, for unconstitutional acts could never live among a people of whom an important percentage were more completely educated.

### In the New World.

In the United States university education has passed into acceptance as the one essential qualification of men or women seeking to occupy a position involving constructive and administrative ability.

It is contended by some thoughtful people that had our own country realised the importance of advanced education twenty years ago Germany would never have possessed such confidence in her superiority, and consequently there would have been no war. On the face of it the contention is reasonable, as such a nation, holding itself so far advantaged over her strongest possible foe, might easily have suffered from " swelled head."

There is no doubt that as a nation we did not appreciate the enormous part that science would play in the Great War. The blunder that sent many of our learned men into the trenches, some to be lost and others to be recalled, is evidence of blindness in this respect. This error of understanding cost us untold wealth and hundreds of thousands of lives, besides placing the Empire in the direst peril.

### Turning the Scales.

Yet can it be denied that, in spite of the initial mistake, our men of science turned the scale in the winning of the war? Hastily collected from the trenches, urged to greater pace in workshop and laboratory, backed by all the resources of the State, our skilled chemists and engineers, our men of theory and men of imagination made the contributions of applied knowledge that told so heavily and brought about the end.

Equally, can it be denied that to the cramped, stereotyped and inelastic education of some in responsible administrative positions we owe much of the incohesion and disorder that nearly brought our nation to disaster? The learning that waxed eloquent on the music of ancient verse, and was silent in research such as that into aniline dyes, nearly proved a costly curriculum to Britain.

### Soldier or Scientist.

The war taught us that the battle is not mainly to the soldier, but to the scientist and to the versatile mind. The great general depended at every turn upon the skill of men of knowledge. It was the mathematician who timed the advance of protecting curtain-fire and measured the fuse of shells, the engineer who devised the tanks and guns, the scientist who winged the aeroplane and dug the trench. It was the neglect of meteorology which cost us many disasters.

And now, in peace times, the captain of industry must turn to the scientist as his chief ally. There is scarcely a trade or calling now but depends upon the skill of chemist, engineer, and mathematician.

Hence the growing need of modern universities. Unless the nation awakens to the paramount importance of this matter, unless it is ready to encourage and intensively develop advanced education in all practicable directions, in another twenty years we shall be as we were in 1914, a nation on the decline physically and educationally when compared with the advancement of other nations. And who can tell but there may again rise the nation, feeling herself superior, and the happenings of 1914 be repeated, but with different results from those in the recent cataclysm?

